# Association between statin use and the risk of colorectal cancer in patients with inflammatory bowel disease: a systematic review and meta-analysis

**DOI:** 10.3389/fimmu.2025.1693342

**Published:** 2026-01-16

**Authors:** Meng Ding, Yang Liu, Ying Zhang, Yunfeng Qiu

**Affiliations:** Department of Endoscopy Center, China-Japan Union Hospital of Jilin University, Changchun, China

**Keywords:** colorectal cancer, incidence, inflammatory bowel disease, meta-analysis, statin

## Abstract

**Background:**

Patients with inflammatory bowel disease (IBD) are at increased risk of colorectal cancer (CRC). Statins exhibit anti-inflammatory and anticancer properties, and although prior meta-analyses have suggested a possible reduction in CRC risk among patients with IBD, the evidence remains limited by small study numbers and methodological constraints.

**Methods:**

We conducted a systematic review and meta-analysis of observational studies comparing CRC incidence between statin users and non-users in IBD populations. PubMed, Embase, and Web of Science databases were searched for relevant studies on May 22, 2025. Data were pooled using a random-effects model, and relative risks (RRs) with 95% confidence intervals (CIs) were calculated. Subgroup and meta-regression analyses were performed to explore potential effect modifiers.

**Results:**

Nine datasets from seven studies involving 639,595 IBD patients were included. Statin use was associated with a significantly reduced CRC risk (RR = 0.77, 95% CI: 0.69–0.87; I² = 27%). The association remained robust in sensitivity analyses and was stronger in high-quality studies (RR = 0.65, 95% CI: 0.54–0.78; I² = 0%). Meta-regression identified follow-up duration as a significant modifier (p = 0.03), and subgroup analysis confirmed that studies with >5 years of follow-up reported a greater risk reduction (p for subgroup difference = 0.02). No significant publication bias was detected (Egger’s test p = 0.35).

**Conclusions:**

Statin use is associated with a lower risk of CRC in patients with IBD, particularly in studies with longer follow-up. These findings support further research on the chemopreventive potential of statins in this high-risk population.

**Systematic review registration:**

https://www.crd.york.ac.uk/prospero/, identifier CRD420251038799.

## Introduction

Colorectal cancer (CRC) is one of the most serious long-term complications of inflammatory bowel disease (IBD), which includes ulcerative colitis (UC) and Crohn’s disease (CD) (1, 2). Patients with IBD face a significantly higher risk of CRC than the general population, particularly those with long-standing colonic inflammation, extensive disease involvement, and early-onset IBD (3, 4). Epidemiological studies have reported that the cumulative risk of CRC in patients with UC may exceed 10% after 20 years of disease duration, with similar elevated risks observed in colonic CD (5, 6). This increased cancer burden is largely attributed to chronic mucosal inflammation, which promotes genomic instability, dysplasia, and neoplastic transformation (7, 8). CRC in IBD patients is also associated with poorer clinical outcomes, more advanced stage at diagnosis, and higher mortality rates compared to sporadic CRC (9). Given the rising prevalence of IBD worldwide and the associated burden of CRC on patient outcomes and healthcare systems, the development of effective chemopreventive strategies is an urgent clinical priority (10, 11).

Statins, or 3-hydroxy-3-methylglutaryl coenzyme A (HMG-CoA) reductase inhibitors, are widely prescribed lipid-lowering agents used in the prevention of cardiovascular disease (12, 13). In addition to their primary role in reducing cholesterol synthesis, statins possess pleiotropic effects, including anti-inflammatory, antioxidant, and immunomodulatory properties (14–16). These effects have drawn increasing attention to the potential use of statins as chemopreventive agents (15). Mechanistically, statins inhibit the mevalonate pathway, which is involved in cell proliferation, survival, and inflammation (17–19). In addition, statins have been shown to reduce tumor growth and suppress key inflammatory and oncogenic pathways relevant to IBD-associated carcinogenesis (20, 21). These findings highlight the potential of statins as a chemoprevention for various cancers in high risk populations.

Growing epidemiologic evidence suggests that statins may reduce the risk of several malignancies, including CRC (22, 23). In the general population, observational studies and meta-analyses have reported modest reductions in CRC incidence among statin users (22, 24). However, despite growing interest, the protective role of statins in IBD-associated CRC requires further clarification (25–31). Two earlier meta-analyses reported a potential reduction in CRC risk among statin users with IBD, but both were constrained by limited study numbers and methodological issues. An early meta-analysis in 2024 (32) included only four studies encompassing 22,250 patients, while a recent meta-analysis in 2025 (33) synthesized seven studies but incorporated one cohort (34) that assessed overall cancer rather than CRC-specific outcomes and did not examine sources of heterogeneity. Moreover, neither of these studies (32, 33) evaluated how study characteristics—such as IBD subtype, geographic region, follow-up duration, or demographic factors—might influence effect estimates. With several large and contemporary cohorts becoming available (27, 29), a comprehensive and methodologically robust meta-analysis is warranted to provide updated CRC-specific estimates and to clarify the influence of study-level factors. Accordingly, we performed a systematic review and meta-analysis aiming to assess the association between statin use and the risk of CRC in patients with IBD.

## Methods

This meta-analysis was conducted in accordance with the Preferred Reporting Items for Systematic Reviews and Meta-Analyses (PRISMA) 2020 guidelines (35, 36) and the methodological standards outlined in the Cochrane Handbook for Systematic Reviews of Interventions (37), ensuring methodological rigor in study selection, data extraction, statistical analysis, and interpretation of results. The completed PRISMA checklist is available in **Supplementary File 1**. The study protocol was prospectively registered in the International Prospective Register of Systematic Reviews (PROSPERO) under the registration number CRD420251038799.

### Search strategy and information sources

To identify eligible studies for this meta-analysis, a comprehensive search was performed across PubMed, Embase, and Web of Science databases. The search strategy incorporated a broad set of keywords and synonyms related to the population, exposure, and outcomes of interest (1): “inflammatory bowel disease” OR “IBD” OR “ulcerative colitis” OR “Crohn disease” OR “Crohn’s disease”; (2) “3-hydroxy-3-methyl-glutarylCoA reductase inhibitor” OR “CS-514” OR “statin” OR specific agents such as “atorvastatin,” “simvastatin,” “fluvastatin,” “lovastatin,” “rosuvastatin,” “pravastatin,” and “pitavastatin”; (3) “colorectal” OR “colorectum” OR “colon” OR “rectal” OR “rectum”; and (4) “neoplasms” OR “carcinoma” OR “cancer” OR “tumor” OR “malignancy” OR “adenoma.” The search was limited to studies involving human participants and articles published in English. To ensure completeness, the reference lists of relevant original studies and review articles were also screened manually. The search encompassed records from database inception through May 22, 2025. A detailed description of the search strategy is provided in **Supplementary File 2**.

### Eligibility criteria

The inclusion criteria for this meta-analysis were defined according to the PICOS framework, encompassing Population, Intervention (Exposure), Comparator, Outcome, and Study Design:

Population: Patients diagnosed with IBD, including CD and/or UC, irrespective of disease severity, duration, or treatment regimen.

Intervention (Exposure): Use of statins following the diagnosis of IBD. Statin exposure was defined based on the methods reported in the original studies, including documented prescriptions for ≥ 3 to ≥ 6 months, prescriptions covering a minimum cumulative defined daily dose, or self-reported regular use.

Comparator: IBD patients who did not receive statin therapy after diagnosis, serving as the control group for each respective study.

Outcome: The incidence of CRC during follow-up. CRC was identified and validated using standardized approaches, such as national cancer registries, International Classification of Diseases (ICD) codes, and expert pathology reviews.

Study Design: Observational studies with longitudinal follow-up, including retrospective or prospective cohort studies, nested case-control studies, and *post-hoc* analyses of clinical trials, were considered eligible. No randomized controlled trials (RCTs) were identified.

Exclusion criteria comprised the following: reviews, editorials, or meta-analyses; preclinical or cross-sectional studies; studies that did not specifically investigate patients with IBD; studies not evaluating statin use as the exposure of interest; and studies without relevant data on CRC incidence. In cases of multiple publications derived from the same population, the study with the largest sample size and most detailed outcome data was selected for inclusion.

### Assessment of study quality

Two reviewers (MD and YL) independently conducted the literature search, screening, quality assessment, and data extraction following a predefined protocol. Duplicate records were identified and removed using EndNote (Version X9, Clarivate™, PA, USA). Titles and abstracts were screened for relevance, and full texts were retrieved for potentially eligible articles. Any disagreements in study selection or data extraction were resolved through discussion; if consensus was not achieved, a third reviewer (YQ) provided arbitration.

The methodological quality of the included studies was assessed using the Newcastle–Ottawa Scale (NOS) (38), which evaluates non-randomized studies based on three major domains: selection of study groups, comparability of cohorts, and ascertainment of outcomes. In the selection domain, emphasis was placed on whether the exposed cohort was representative of the target IBD population, particularly favoring studies that enrolled patients consecutively or through random sampling. Ascertainment of statin exposure was considered adequate if use was documented via clinical records or prescription data, rather than self-reports. For outcome assessment, studies utilizing centralized pathology review or national cancer registries for the diagnosis of CRC were judged to have high methodological rigor, whereas reliance solely on ICD codes was deemed less robust. The follow-up period was also evaluated, with duration of at least five years considered sufficient to detect the development of CRC. For comparability, studies were assessed based on whether they adjusted for key confounders such as age, sex, IBD subtype, comorbidities, and medication use. A maximum of nine points could be awarded, and studies scoring seven or more were classified as high quality.

### Data collection

Data extraction was performed independently by the same two reviewers using a predesigned Microsoft Excel spreadsheet, ensuring standardization and reproducibility. Extracted data included publication details (first author, year of publication, country, and study design), patient demographics (IBD subtype, sample size, age, and sex distribution), exposure characteristics (definition of statin use, timing, duration, and number of users), follow-up duration, and the method used to validate CRC outcomes. In addition, the number of patients who developed CRC and the covariates included in adjusted statistical models were recorded. All extracted data were cross-verified, and discrepancies were resolved through consensus.

### Statistical analysis

The association between statin use and the risk of CRC in patients with IBD was assessed by pooling relative risks (RRs) with corresponding 95% confidence intervals (CIs). When RRs were not directly reported, they were calculated from available 95% CIs or p-values and log-transformed to stabilize variance. The log-transformed RRs and their standard errors were then synthesized using the inverse-variance method (37). For studies reporting odds ratios (ORs), estimates were converted to RRs using the formula: RR = OR/([1 − pRef] + [pRef × OR]), where pRef denotes the incidence of CRC in the reference (non-statin) group (39). Heterogeneity across studies was assessed using the Cochrane Q test and quantified with the I² statistic, with I² > 50% considered indicative of substantial heterogeneity (40). Given the expected diversity in patient characteristics, definitions of statin exposure, and CRC outcome validation, a random-effects model was applied to account for between-study variability (37).

To examine the robustness of the pooled estimates, sensitivity analyses were conducted by restricting the analysis to studies rated as high quality (NOS score ≥ 7) and by sequentially omitting individual studies (37). Furthermore, univariate meta-regression analyses were performed to explore the influence of study-level characteristics, treated as continuous variables, on the pooled effect estimates (37). These characteristics included the proportion of patients with Crohn’s disease (reflecting IBD subtype), mean age, proportion of male participants, follow-up duration, and NOS scores. Predefined subgroup analyses were also conducted to investigate the potential modifying effects of key study features, including geographic location, proportion of patients with CD, mean age, sex distribution, follow-up duration, and the method of CRC outcome validation. For the purposes of subgroup stratification, continuous variables were dichotomized based on their median values.

Potential publication bias was evaluated through visual inspection of funnel plot symmetry and statistically tested using Egger’s regression test (41). All statistical analyses were conducted using Review Manager (RevMan), Version 5.1 (Cochrane Collaboration, Oxford, UK) and Stata, Version 17.0 (StataCorp, College Station, TX, USA). The certainty of evidence was appraised according to the Grading of Recommendations Assessment, Development, and Evaluation (GRADE) approach, incorporating five domains: risk of bias (limitations in study design and conduct), inconsistency (heterogeneity of results), indirectness (applicability of the evidence), imprecision (width of confidence intervals), and risk of publication bias (42). A Summary of Findings table was generated in accordance with the Cochrane Handbook (37).

## Results

### Screening and selection of eligible studies

**Figure 1:**
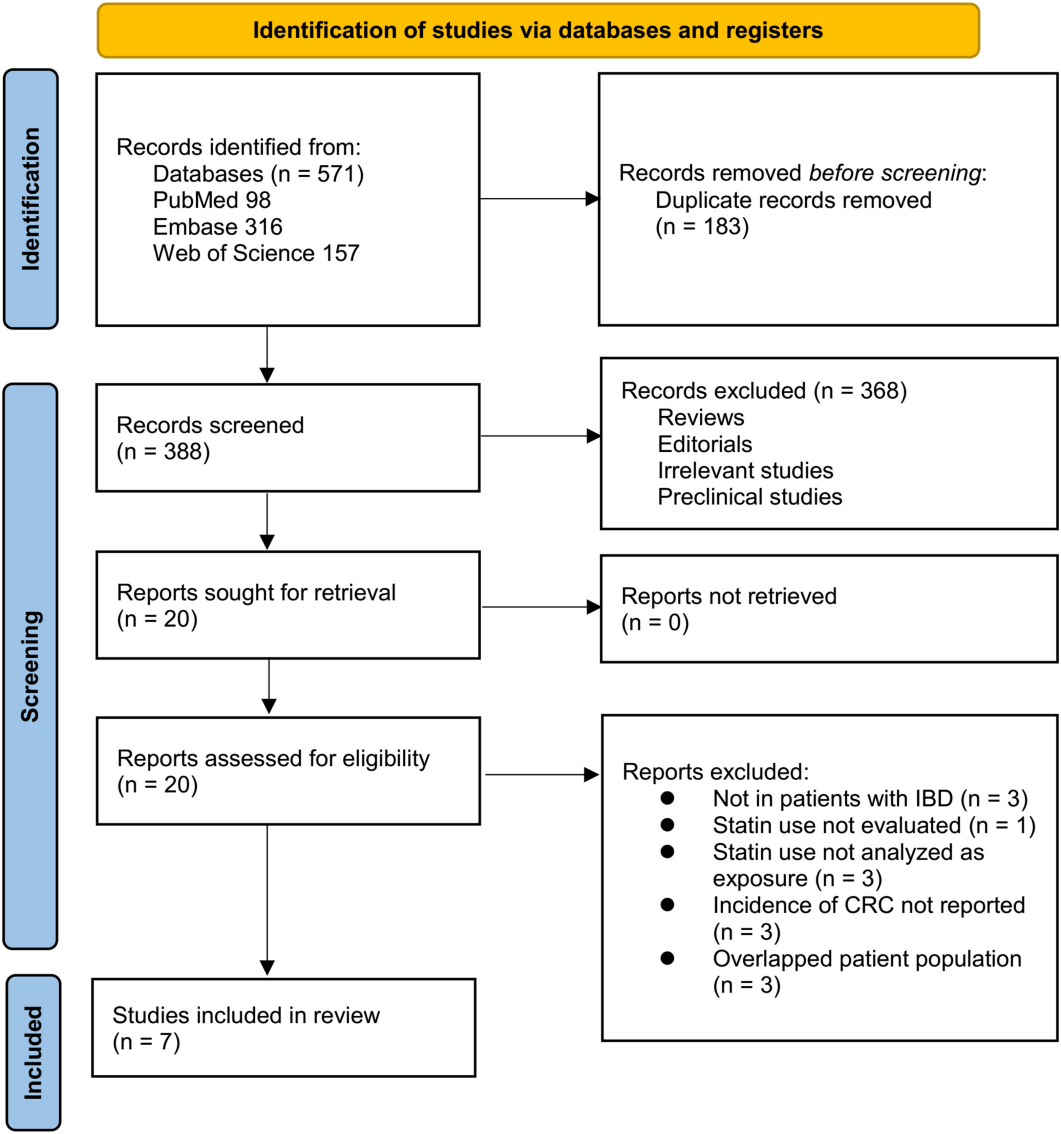
PRISMA flow diagram of study selection. Flow chart illustrating the selection process of eligible studies for the meta-analysis, including the number of records identified, screened, assessed for eligibility, and included, with reasons for exclusion at each stage.

**Figure 1** illustrates the detailed study selection process. A total of 571 potentially relevant records were identified through searches of the three databases. After removing 183 duplicates, 388 unique records remained for title and abstract screening. Of these, 368 were excluded for not meeting the eligibility criteria or lacking relevance to the objectives of this meta-analysis. The full texts of the remaining 20 articles were independently assessed by two reviewers. Thirteen studies were excluded based on predefined exclusion criteria, as detailed in **Figure 1**. Consequently, seven studies met all inclusion criteria and were included in the final quantitative synthesis (25–31).

### Characteristics of included studies

**Table 1** presents the key characteristics of the seven eligible observational studies included in this meta-analysis, encompassing a total of 635,595 patients with inflammatory IBD (25–31). Since two of them reported stratified results: one study (29) provided separate data for patients with CD and UC, while another study (31) reported sex-specific outcomes, these were treated as four distinct analytical datasets, resulting in nine datasets included in the quantitative synthesis. The included studies spanned multiple geographical regions, with populations from the United States, Israel, Sweden, Hong Kong (China), and South Korea. One study employed a prospective cohort design (31), while the remaining six were retrospective cohort (26–30) or nested case-control studies (25). Publication years ranged from 2011 to 2023. Most studies included mixed IBD populations (CD and UC) (25–28, 31), although three datasets focused specifically on either CD (29) or UC (29, 30). The average participant age ranged from 41.0 to 70.0 years, and the proportion of men varied between 46.5% and 60.7%. Statin exposure was defined across studies based on self-reported use (25), documented prescriptions or clinical records (26–31), with minimum exposure durations ranging from 30 days to five years. A total of 112,650 (17.7%) patients were statin users. Follow-up durations ranged from 3.9 to 10 years, with most studies meeting the threshold of at least five years required for adequate CRC risk evaluation (25–27, 29–31). The methods used to validate CRC diagnoses varied. High-confidence methods included pathology review by expert gastrointestinal pathologists (25, 28) and national cancer registry linkage (30, 31). Other studies (26, 27, 29) relied on administrative coding (ICD-9 or ICD-10) for outcome identification. Multivariable adjustment was performed in most studies to control for potential confounders such as age, sex, comorbidities, IBD subtype and medications, healthcare utilization, and inflammatory burden. Only one study (27) adjusted for age and sex alone, while the remainder adjusted for broader clinical and demographic variables.

**Table 1:** Characteristics of the included cohort studies.

Study	Country	Study design	Diangosis	Sample size	CD (%)	Mean age (years)	Men (%)	Definition of statin sue	No. of patients with statin use	Median follow-up duration (years)	Methods for validation of CRC	No. of patients with CRC	Variables adjusted
Samadder 2011	Israel	NCC	CD and UC	60	6.7	70	51.4	Self-reported regular use of any statin for at least five years	6	5	Centralized pathology review by a single expert pathologist	39	Age, sex, ethnicity, vegetable consumption, history of CRC in first-degree relative, sports participation and smoking status
Ananthakrishnan 2016	USA	RC	CD and UC	11001	49.4	45.1	47.1	≥1 electronic prescription ≥6 months before CRC or follow-up end	1376	9	ICD-9 codes	317	Age, sex, race, smoking, inflammatory markers, PSC, colonoscopy history, follow-up time, immunomodulator and anti-TNF use, healthcare utilization, and propensity score
So 2017	Hongkong (China)	RC	CD and UC	2622	38.9	49	59.4	At least one continuous statin prescription lasting ≥6 months	278	10	ICD-9 codes	19	Age and sex
Shah 2019	USA	RC	CD and UC	642	47.2	41.3	49.1	Any documented statin use for ≥3 months based on prescriptions or clinical documentation	57	3.9	Pathology reports from surveillance colonoscopies, reviewed and confirmed by expert gastrointestinal pathologists	6	Age, sex, PSC, IBD duration, inflammation score, colonoscopy frequency, and immunosuppressive therapies
Hamid 2023 CD	USA	RC	CD	299732	100	NR	46.5	Documented use of lipophilic statins	40020	5	ICD-10 codes	NR	Age, sex, comorbidities, and IBD treatments
Hamid 2023 UC	USA	RC	UC	279803	0	NR	50.1	Documented use of lipophilic statins	58205	5	ICD-10 codes	NR	Age, sex, comorbidities, and IBD treatments
Sun 2023	Sweden	PC	CD and UC	10546	30.1	62.6	56.9	A prescription covering ≥30 cumulative defined daily doses	5273	5.6	Swedish Cancer Register	160	Age, sex, IBD subtype, calendar period, socioeconomic status, comorbidities, and co-medications
Oh 2023	South Korea	RC	UC	35189	0	41	60.7	Prescription of statins (any type) for more than 30 days	7435	6	Korea Central Cancer Registry	122	Age, sex, comorbidities, and treatment for UC

CD, Crohn’s disease; UC, ulcerative colitis; CRC, colorectal cancer; NCC, nested case-control; RC, retrospective cohort; PC, prospective cohort; ICD-9, International Classification of Diseases, 9th Revision; ICD-10, International Classification of Diseases, 10th Revision; PSC, primary sclerosing cholangitis; NR, not reported.

### Study quality evaluation

As shown in **Table 2**, the methodological quality of the included studies was assessed using the NOS. Total NOS scores ranged from 6 to 9. Most studies adequately selected comparison cohorts and documented exposure using reliable sources (26–31). However, representativeness of the exposed cohorts was limited in several studies due to selection from specific clinical populations (27, 31). Overall, six studies were rated as high quality (25–28, 30, 31), while one study scored 6 (29), reflecting moderate quality primarily due to limited outcome ascertainment and follow-up duration.

**Table 2:** Study quality evaluation of the included studies via the Newcastle-Ottawa Scale.

Study	Representativeness of the exposed cohort	Selection of the non-exposed cohort	Ascertainment of exposure	Outcome not present at baseline	Control for age and sex	Control for other confounding factors	Assessment of outcome	Enough long follow-up duration
Samadder 2011	0	1	0	1	1	1	1	1
Ananthakrishnan 2016	0	1	1	1	1	1	0	1
So 2017	1	1	1	1	1	0	1	1
Shah 2019	0	1	1	1	1	1	1	0
Hamid 2023 CD	0	1	1	1	1	1	0	0
Hamid 2023 UC	0	1	1	1	1	1	0	0
Sun 2023	1	1	1	1	1	1	1	1
Oh 2023	0	1	1	1	1	1	1	1

### Association between statin use and the risk of CRC

**Figure 2:**
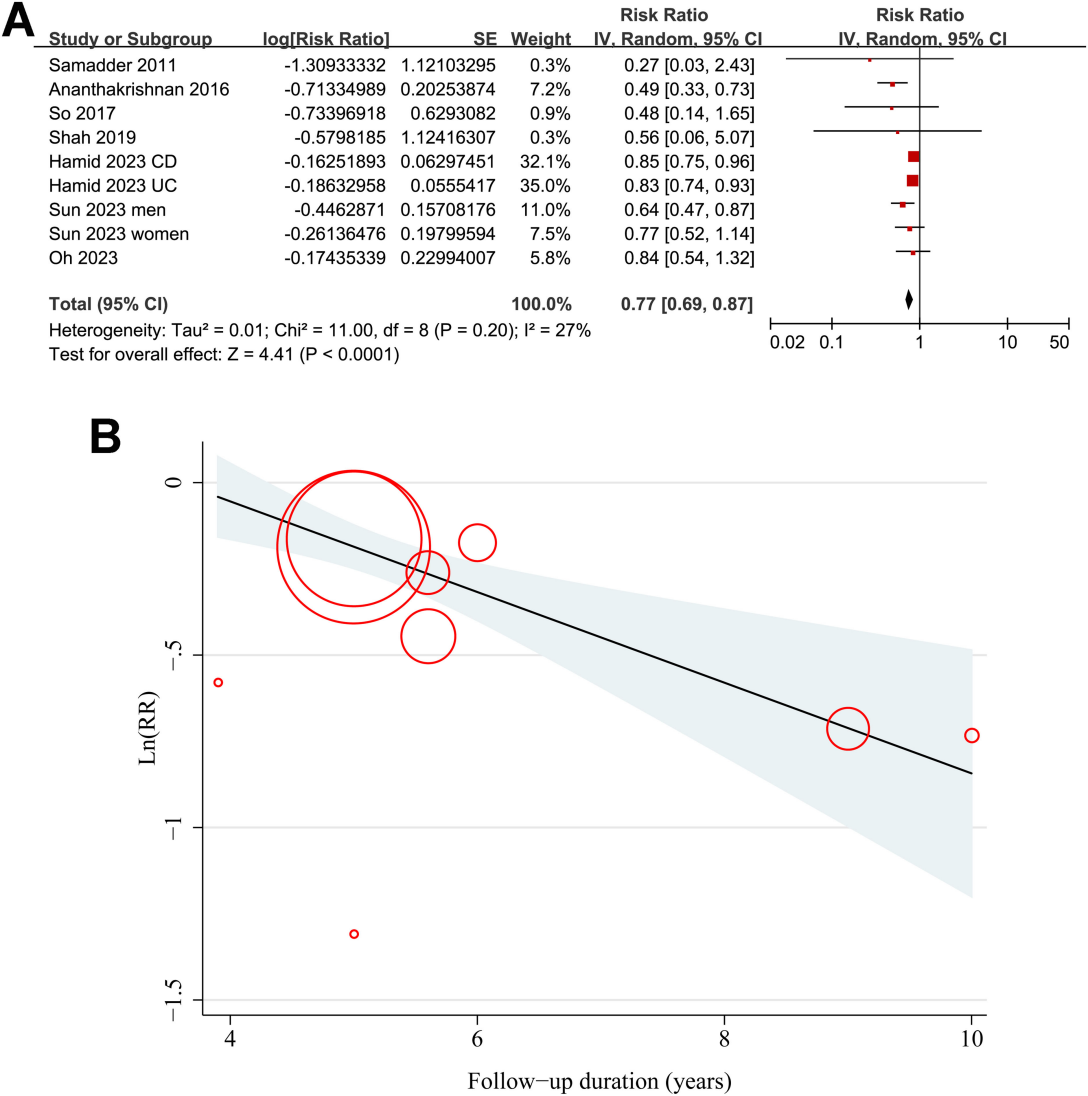
Overall and meta-regression results of the association between statin use and the risk of colorectal cancer in patients with inflammatory bowel disease. **(A)** Forest plot summarizing the pooled relative risk (RR) and 95% confidence intervals (CIs) for the association between statin use and CRC risk across nine datasets. **(B)** Bubble plot of univariate meta-regression showing the modifying effect of follow-up duration on the pooled effect estimate.

**Figure 3:**
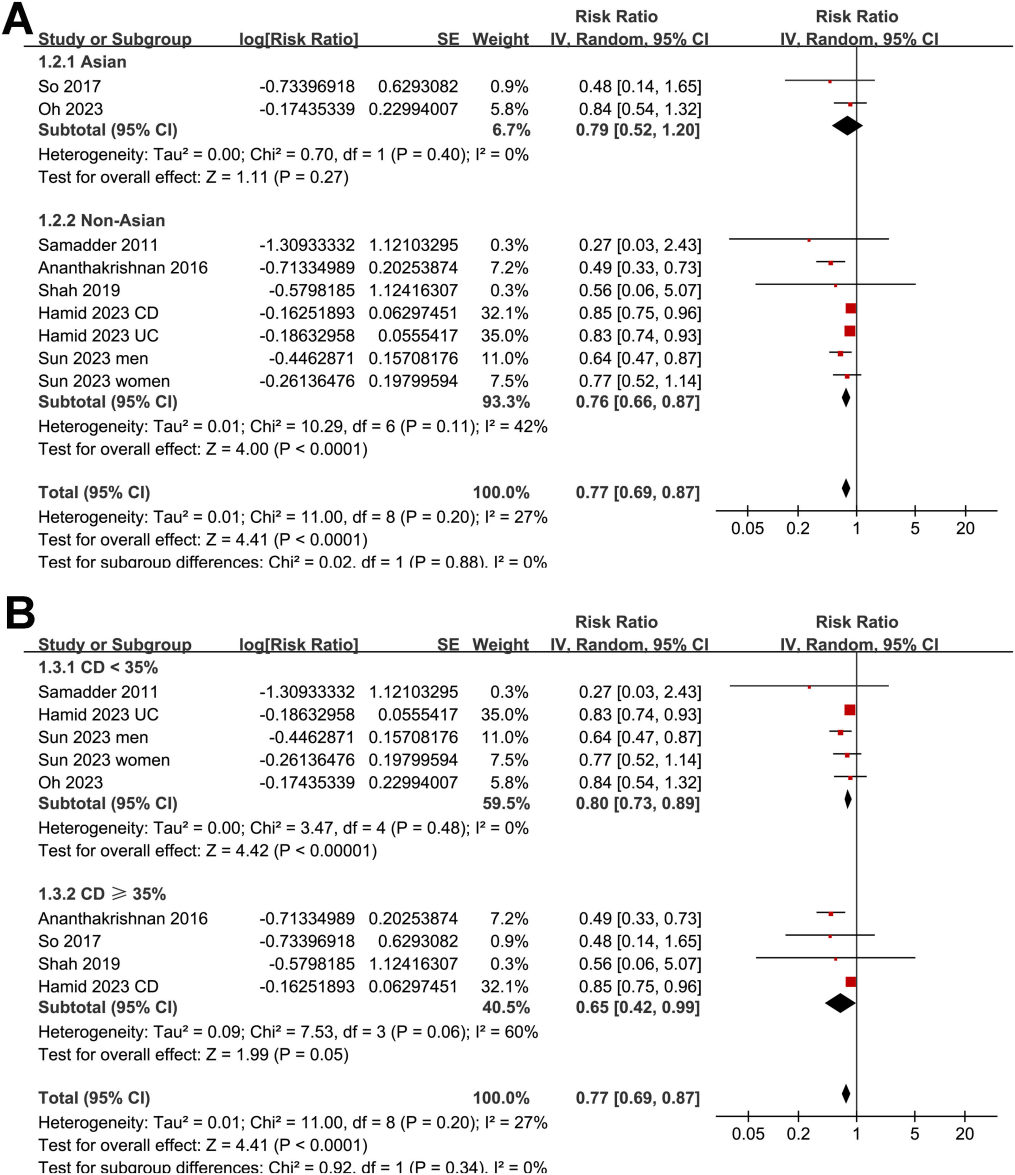
Subgroup analyses by study region and subtype of IBD. **(A)** Forest plot showing the association between statin use and CRC risk stratified by geographic location of the study population. **(B)** Forest plot showing the association between statin use and CRC risk stratified by proportions of CD of the included cohorts.

**Figure 4:**
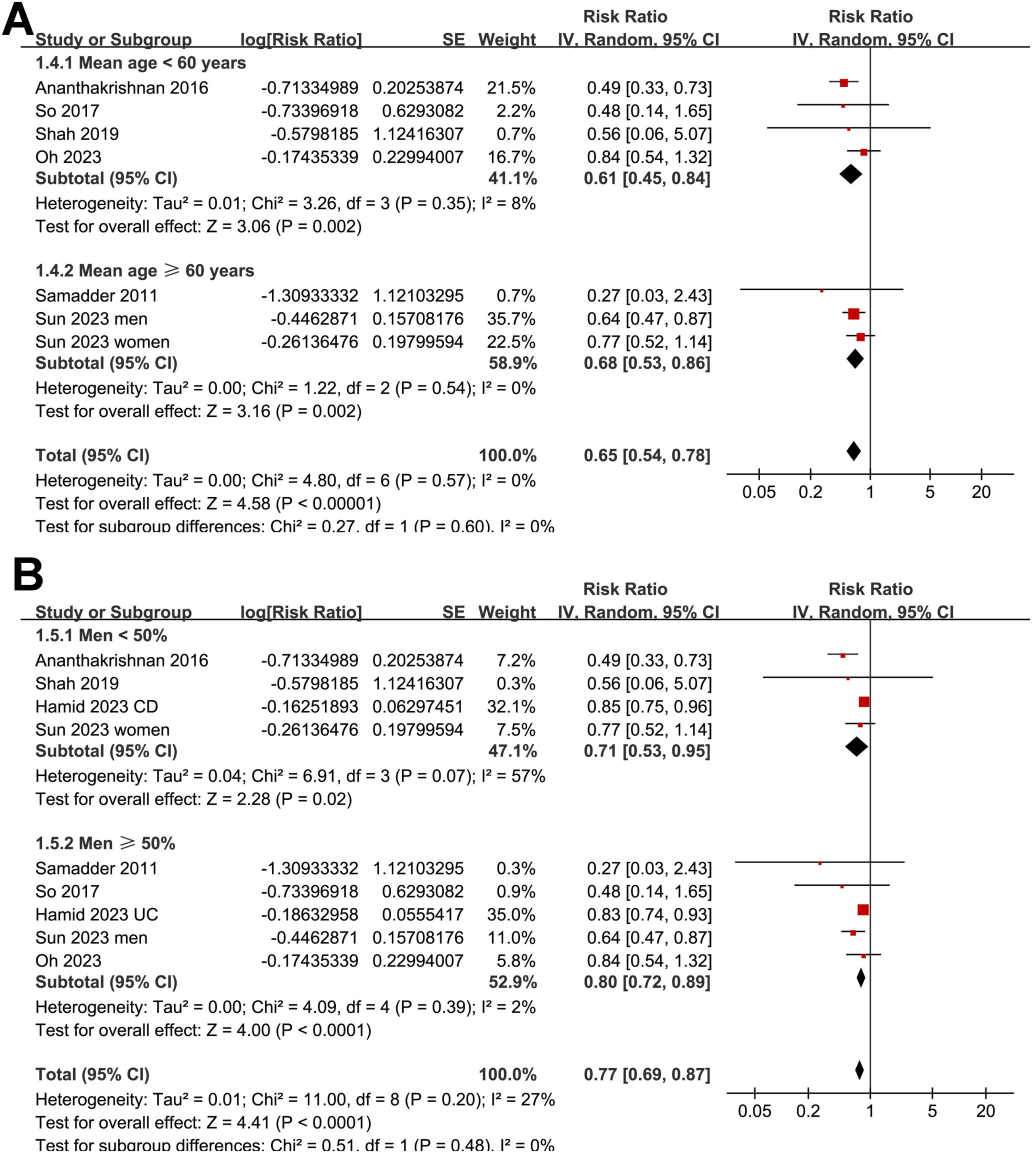
Subgroup analyses by mean ages and sex distribution of patients with IBD. **(A)** Forest plot showing the association between statin use and CRC risk stratified by mean ages of the study population. **(B)** Forest plot showing the association between statin use and CRC risk stratified by proportions of men of the included cohorts.

**Figure 5:**
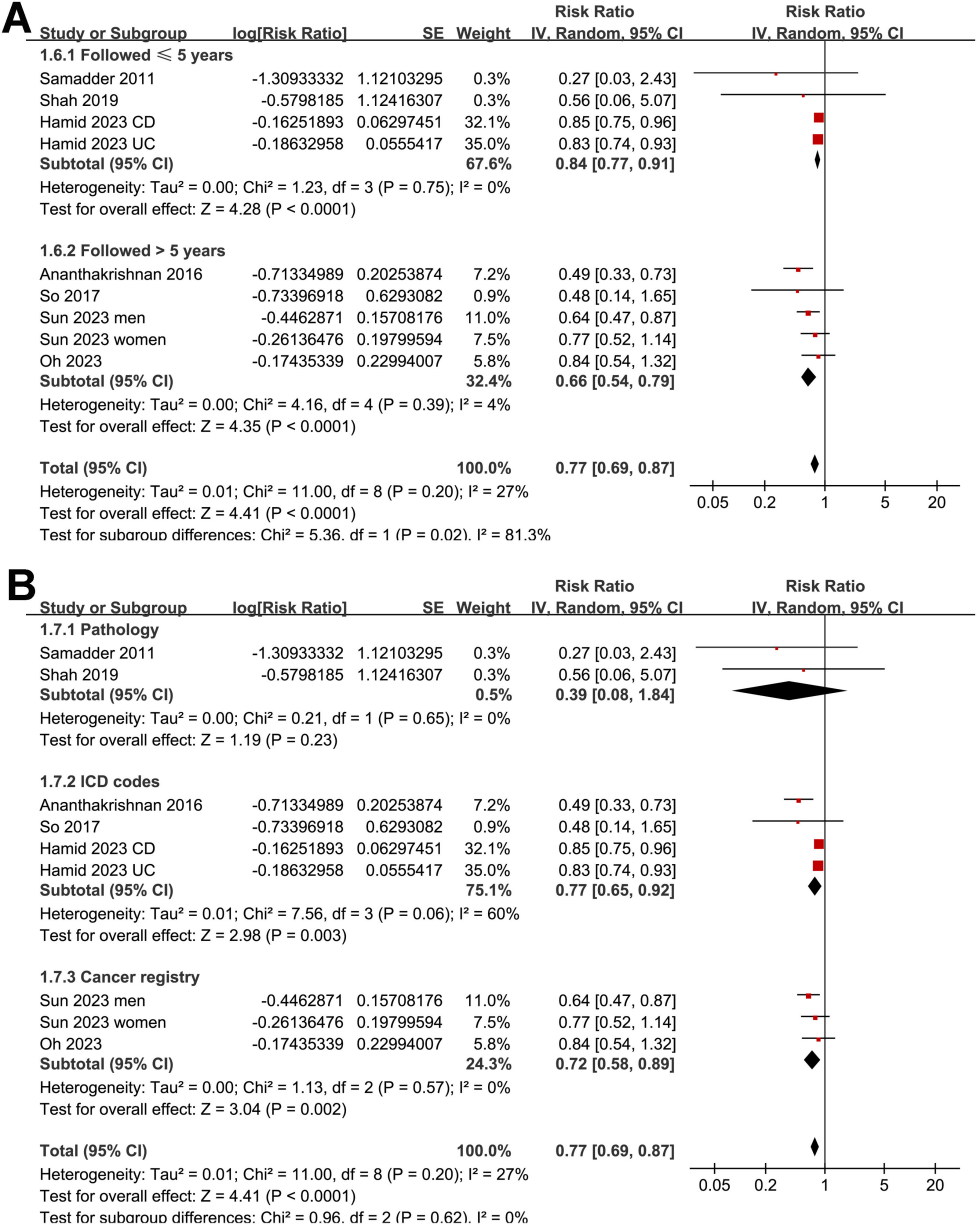
Subgroup analyses by follow-up durations and methods for validating CRC cases. **(A)** Forest plot showing the association between statin use and CRC risk stratified by mean follow-up durations. **(B)** Forest plot showing the association between statin use and CRC risk stratified by methods for the validation of CRC diagnosis in each study.

As illustrated in **Figure 2A**, the meta-analysis of nine datasets derived from seven observational studies (25–31) involving 639,595 patients with IBD demonstrated a significant inverse association between statin use and the risk of CRC. Patients with IBD who used statins after diagnosis had a 23% lower risk of developing CRC compared to non-users (RR = 0.77, 95% CI: 0.69–0.87, p < 0.001), with moderate heterogeneity across studies (I² = 27%). The certainty of evidence by the GRADE assessment is shown in **Table 3**. The certainty of evidence was rated as moderate because the small number of included studies limited our ability to evaluate publication bias and reduced confidence in the precision of the pooled estimates. Robustness of the findings was confirmed through multiple sensitivity analyses (**Table 4**), which showed that the exclusion of any single dataset did not materially change the overall results. Restricting the analysis to high-quality studies (NOS ≥ 7) (25–28, 30, 31) yielded an even stronger association (RR = 0.65, 95% CI: 0.54–0.78, p < 0.001) with no evidence of heterogeneity (I² = 0%). Univariate meta-regression (**Table 5**, **Figure 2B**) indicated that longer follow-up durations were associated with greater protective effects of statins (p = 0.03), while other study-level characteristics, including patient age, sex distribution, disease subtype, and NOS score, did not significantly influence effect size. Subgroup analyses (**Figures 3**–**5**) confirmed the consistency of the association across geographic regions, proportions of CD within cohorts, mean ages and the proportions of men of the included patients, and methods used to validate CRC diagnosis (p for subgroup difference all > 0.05). Notably, a statistically significant subgroup difference was detected based on follow-up duration (p for subgroup difference = 0.02; **Figure 5A**), indicating that studies with longer follow-up (> 5 year) reported stronger protective effects of statins. These findings are consistent with the results of the meta-regression analysis, indicating that variation in follow-up duration may represent a key contributor to the observed heterogeneity.

**Table 3:** Summary of findings.

Association between statin use and the risk of colorectal cancer in patients with inflammatory bowel disease				
Patients: patients with inflammatory bowel disease; Exposure: with a statin use after the diagnosis of IBD; Comparison: without a statin use after the diagnosis of IBD;				
Outcomes	Relative effect (95% CI)	Patient number (datasets)	Certainty of the evidence (GRADE)	Comments
Overall survival	RR 0.77 (0.69 to 0.87)	639,595 (9 comparisons from 7 studies)	Moderate ^a^	Statin use was associated with a 23% reduction of CRC risk in patients with IBD

GRADE Working Group grades of evidence; High certainty: We are very confident that the true effect lies close to that of the estimated effect. Moderate certainty: We are moderately confident in the estimated effect. The true effect is likely to be close to the estimated effect, but there is a possibility that it is substantially deferent. Low certainty: Our confidence in the estimated effect is limited: The true effect may be substantially different from the estimated effect. Very low certainty: We have very little confidence in the estimated effect. The true effect is likely to be substantially different from the estimated effect.

CI, confidence interval; RR relative risk; CART-T, IBD, inflammatory bowel disease; CRC, colorectal cancer;

a Downgraded one point as possible risk of publication bias due to the limited number of studies included in the meta-analysis;

**Table 4:** Sensitivity analyses.

	Meta-analysis for the association statin use and risk of CRC in patients with IBD			
Dataset omitted	RR [95% CI]	P for effect	I^2^	P for Cochrane Q test
Studies with NOS < 7	0.65 [0.54, 0.78]	< 0.001	0%	0.57
Samadder 2011	0.77 [0.69, 0.87]	< 0.001	30%	0.19
Ananthakrishnan 2016	0.82 [0.76, 0.88]	< 0.001	0%	0.68
So 2017	0.77 [0.69, 0.87]	< 0.001	32%	0.17
Shah 2019	0.77 [0.68, 0.87]	< 0.001	36%	0.14
Hamid 2023 CD	0.72 [0.61, 0.85]	< 0.001	29%	0.20
Hamid 2023 UC	0.72 [0.60, 0.86]	< 0.001	33%	0.17
Sun 2023 men	0.80 [0.71, 0.89]	< 0.001	20%	0.27
Sun 2023 women	0.76 [0.67, 0.87]	< 0.001	36%	0.14
Oh 2023	0.76 [0.67, 0.86]	< 0.001	36%	0.14

IBD, inflammatory bowel disease; CRC, colorectal cancer; NOS, Newcastle-Ottawa Scale; RR, relative risk; CI, confidence interval; CD, Crohn’s disease; UC, ulcerative colitis;

**Table 5:** Results of univariate meta-regression analysis.

Variables	RR for the incidence of CRC		
	Coefficient	95% CI	P values
CD (%) of the included patients	0.00019	-0.00548 to 0.00587	0.94
Mean age (years)	0.0071	-0.0194 to 0.0335	0.55
Men (%)	-0.0019	-0.0092 to 0.0054	0.55
Follow-up duration (years)	-0.13	-0.24 to -0.02	0.03
NOS	-0.075	-0.176 to 0.025	0.12

CRC, colorectal cancer; CD, Crohn’s disease; RR, relative risk; CI, confidence interval; NOS, Newcastle-Ottawa Scale.

### Publication bias

**Figure 6:**
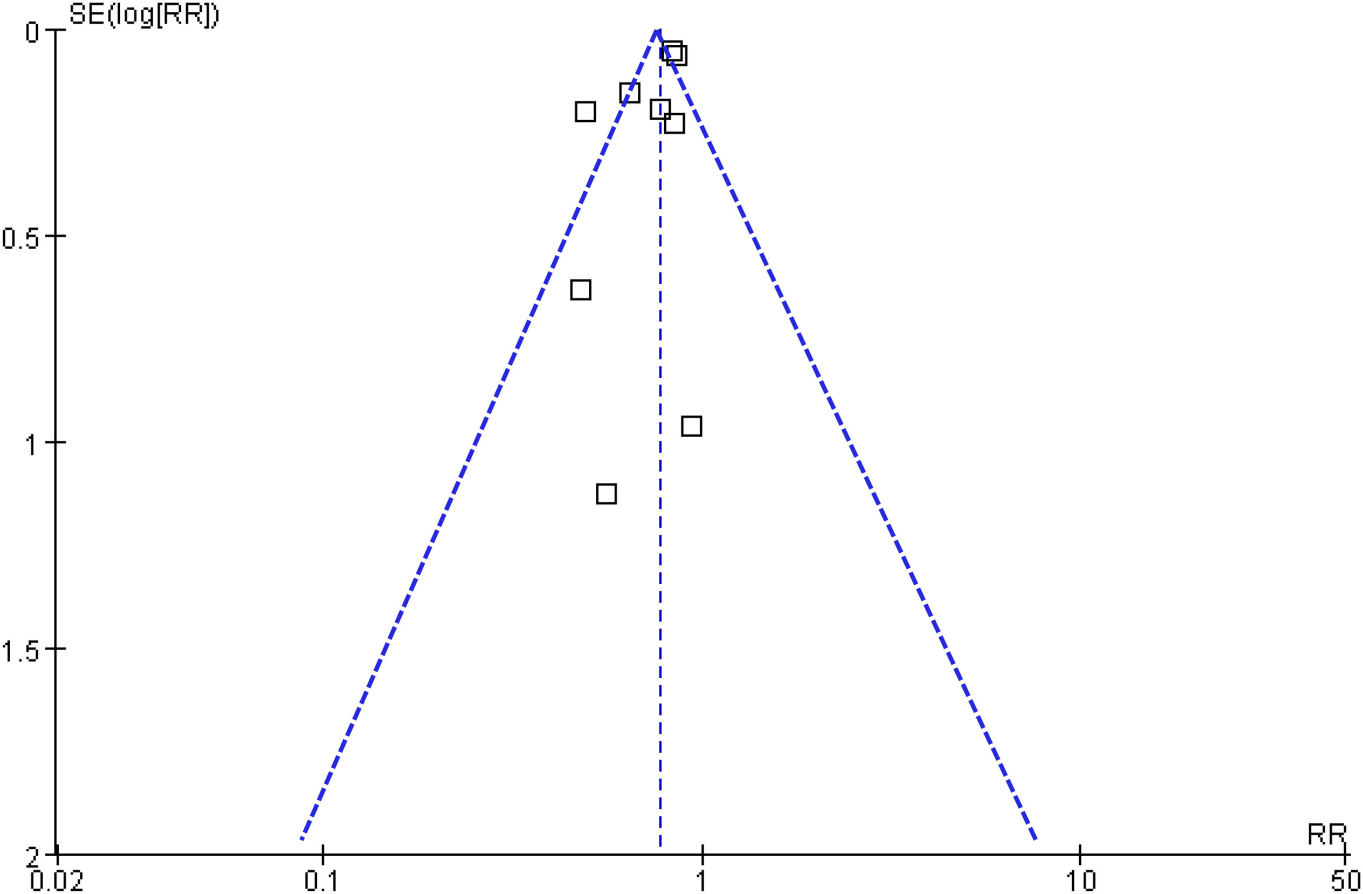
Funnel plot for publication bias assessment. Funnel plot examining the symmetry of effect estimates from the included studies. Visual inspection and Egger’s test (p = 0.35) suggest a low risk of publication bias, although interpretation should be cautious due to the limited number of datasets.

Visual inspection of the funnel plot assessing the association between statin use and CRC risk in patients with IBD revealed a symmetrical distribution, indicating a low likelihood of publication bias (**Figure 6**). This was further supported by Egger’s regression test (p = 0.35). Nevertheless, these findings should be interpreted with caution due to the limited number of datasets included in the analysis.

## Discussion

This systematic review and meta-analysis of nine datasets from seven observational studies involving over 630,000 patients with IBD provides compelling evidence that statin use is associated with a significantly reduced risk of CRC. The pooled results demonstrated a 23% relative risk reduction in CRC among statin users compared to non-users, and this association remained robust across a variety of sensitivity and subgroup analyses. Notably, a stronger protective effect was observed in studies with longer follow-up durations and higher methodological quality, and follow-up length was identified as a significant modifier in the meta-regression analysis. These findings offer a comprehensive synthesis of the current literature and highlight the potential role of statins as a chemopreventive strategy in patients with IBD, a population at increased risk for inflammation-driven CRC.

**Figure 7:**
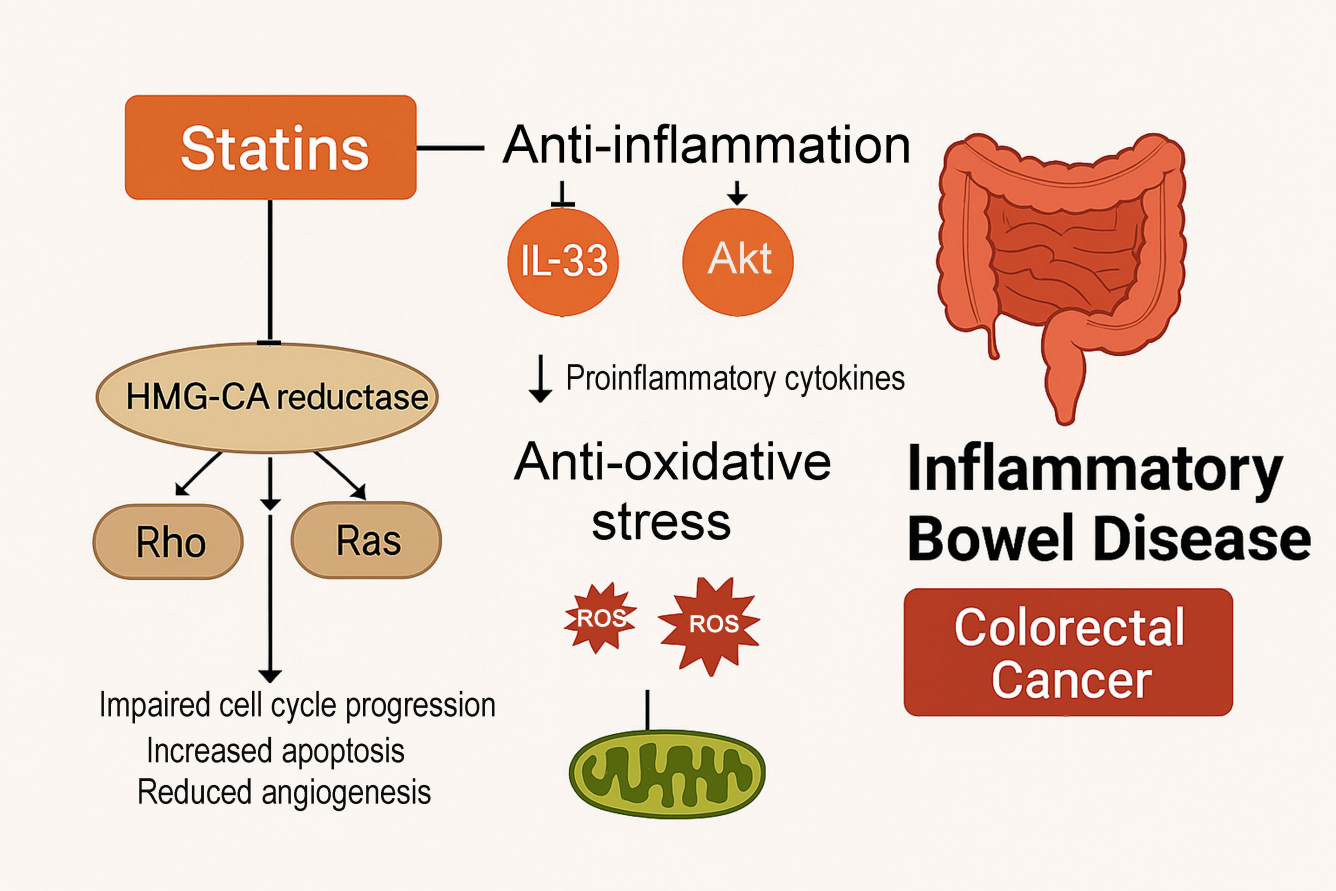
Illustration of the proposed molecular mechanisms by which statins may reduce CRC risk in patients with IBD. Statins inhibit HMG-CoA reductase, a key enzyme in the mevalonate pathway, thereby suppressing the activity of downstream signaling molecules such as Ras and Rho. This inhibition leads to impaired cell cycle progression, increased apoptosis, and reduced angiogenesis. Statins also exhibit anti-inflammatory and antioxidative effects by downregulating pro-inflammatory cytokines (e.g., IL-33), regulation Akt, and reducing reactive oxygen species. These mechanisms may collectively attenuate chronic inflammation and carcinogenic processes in the colonic epithelium of patients with IBD, thereby lowering the risk of CRC.

The observed association may be supported by several biological mechanisms (**Figure 7**). Statins inhibit HMG-CoA reductase, a key enzyme in the mevalonate pathway, which regulates cholesterol biosynthesis and produces intermediates essential for post-translational modification of small GTP-binding proteins (e.g., Ras, Rho, and Rac) (15, 43, 44). These proteins play crucial roles in cell signaling, proliferation, survival, and motility—processes that are frequently dysregulated in oncogenesis (45–47). Inhibiting this pathway leads to impaired cell cycle progression, increased apoptosis, and reduced angiogenesis, all of which may contribute to tumor suppression. Additionally, statins exhibit anti-inflammatory properties by downregulating pro-inflammatory cytokines (e.g., interleukin 33) (48), and regulating Akt signaling (49)—two key signaling pathways implicated in chronic inflammation and colorectal tumorigenesis in IBD (50). Statins have also been shown to reduce oxidative stress by lowering reactive oxygen species (51), which are known to promote DNA damage and tumor initiation. These mechanisms are particularly relevant in the IBD setting, where chronic colonic inflammation drives epithelial dysplasia and neoplastic transformation (52, 53).

The consistency of the inverse association across multiple analyses supports the robustness of the findings. In sensitivity analyses, the exclusion of any single study did not materially change the overall effect estimate. Furthermore, when restricted to high-quality studies (NOS ≥ 7), the effect size was stronger and heterogeneity disappeared, suggesting that the quality of study design and data ascertainment influences the observed associations. The meta-regression analysis revealed that longer follow-up durations were significantly associated with greater reductions in CRC risk, indicating that statins may require sustained use over time to confer meaningful protective effects. Subgroup analyses confirmed that the association between statin use and reduced CRC risk persisted across various study regions, sex distributions, mean ages, and proportions of patients with CD. Importantly, subgroup analysis by follow-up duration revealed a statistically significant difference, with studies reporting follow-up periods of more than five years showing more pronounced risk reductions. This suggests a potential time-dependent effect of statins in preventing inflammation-associated carcinogenesis, which is consistent with previous findings evaluating the chemopreventive potential of statins for cancer in patients with liver disease, interstitial lung disease, and pulmonary fibrosis (54, 55). Although individual-level duration of statin exposure was not uniformly available, this time-dependent pattern suggests that sustained statin use may be required to achieve meaningful chemopreventive benefits, consistent with duration-dependent cancer reduction reported in other populations. For example, Ren et al. (56) demonstrated a duration-dependent reduction in both cancer incidence and cancer-related mortality among statin users with heart failure, with the strongest benefit observed after more than six years of continuous use. It should be noted that exposure criteria reported by individual studies in this meta-analysis (e.g., ≥ 3–6 months or cumulative defined doses) were designed only to define statin users and do not reflect actual treatment duration; therefore, these data could not be synthesized into an interpretable pooled duration range.

Additionally, studies employing validated CRC outcome measures (e.g., pathology reports) seemed to show stronger associations than those using administrative coding alone (ICD codes), highlighting the importance of accurate outcome ascertainment.

This study has several notable strengths. It represents the most comprehensive and up-to-date synthesis of available evidence on the relationship between statin use and CRC risk in IBD patients, incorporating a large sample size across multiple countries and populations. Only longitudinal observational studies were included, allowing for temporal assessment of statin exposure prior to CRC development. All included studies employed multivariable adjustment, which improves the reliability of effect estimates by accounting for known confounders such as age, sex, comorbidities, medication use, and surveillance practices. Furthermore, multiple complementary analyses—including sensitivity, subgroup, and meta-regression—were conducted to assess the stability of findings and explore potential sources of heterogeneity, enhancing the methodological rigor of this review. Nevertheless, several limitations must be acknowledged. First, the majority of included studies were retrospective in design, which inherently introduces the risk of selection bias, misclassification of exposures and outcomes, and residual confounding (57). Statin use was often identified from administrative data or self-report, which may not fully capture adherence or over-the-counter use. Although most studies adjusted for key clinical and demographic variables, unmeasured confounding—such as dietary patterns, physical activity, family history of cancer, or genetic risk factors (58, 59)—may still influence the observed associations. Moreover, this meta-analysis relied on study-level rather than individual patient-level data, precluding detailed exploration of how factors such as IBD activity, disease location, or treatment history may modify the effect of statins. Additionally, due to data limitations, we were unable to assess the influence of statin type (e.g., lipophilic *vs*. hydrophilic), dosage, or duration of use on CRC risk. Definitions of statin exposure varied substantially across studies (from short-term use to multi-year duration), and because individual-patient data were unavailable, we were unable to perform stratified analyses based on exposure duration, which may have contributed to residual heterogeneity. On the other hand, although follow-up duration was identified as a key contributor to heterogeneity via study level meta-regression and subgroup analysis, other potential modifiers—such as statin type, dose, and cumulative exposure—were inconsistently reported and could not be examined, leaving some heterogeneity unexplained. Although subgroup analyses were conducted based on IBD subtype proportions, the effect of statins may differ between patients with UC and CD due to distinct pathophysiologic mechanisms (60), and this could not be adequately explored. Furthermore, the relatively small number of eligible studies limits the power of publication bias assessments; while funnel plot inspection and Egger’s test did not indicate asymmetry, the potential for publication bias remains and should be interpreted cautiously. Finally, the observational nature of the included studies precludes any causal inference, and the potential for indication bias (e.g., less severe IBD individuals being more likely to receive statins) cannot be ruled out.

Despite these limitations, the findings of this meta-analysis have meaningful clinical implications. Given the established safety profile and widespread use of statins for cardiovascular prevention, their potential chemopreventive benefit in patients with IBD could be of considerable value—particularly in older patients or those with concurrent cardiovascular risk factors. However, before statins can be recommended for cancer prevention in clinical guidelines, further research is needed to confirm these findings in prospective settings. Future studies should aim to clarify the optimal type, dose, and duration of statin use, and investigate whether certain patient subgroups derive greater benefit. Importantly, well-designed prospective cohort studies and RCTs may be considered to address residual confounding and determine causality. Additionally, mechanistic studies exploring the interaction between statins and the inflamed colonic microenvironment in IBD could provide further insight into the biological basis of these associations. However, at current stage, it has to be mentioned that because all included studies were observational, our findings demonstrate an association rather than a causal effect. Residual confounding—such as healthier-user effects, differences in disease severity, health-seeking behavior, or concurrent therapies—may have contributed to the observed risk reduction, and therefore the results should be interpreted cautiously.

## Conclusions

In conclusion, this meta-analysis suggests that statin use is associated with a significantly reduced risk of colorectal cancer among patients with inflammatory bowel disease, particularly in studies with longer follow-up. These findings support the hypothesis that statins may offer chemopreventive benefits in this high-risk population. While current evidence is encouraging, future prospective studies and clinical trials are warranted to validate these observations and inform the potential integration of statins into CRC prevention strategies for patients with IBD.

## Data Availability

The original contributions presented in the study are included in the article/**Supplementary Material**. Further inquiries can be directed to the corresponding authors.
