# Supplementary material for: Association between statin use and the risk of colorectal cancer in patients with inflammatory bowel disease: a systematic review and meta-analysis
Source: Front Immunol. 2026 Jan 16;16:1693342. doi: 10.3389/fimmu.2025.1693342 (PMC12855103; doi:10.3389/fimmu.2025.1693342)
Supplement: Supplementary file 1 [file SupplementaryFile1.docx]

**Supplemental File 1** Detailed Search strategy for each database

**PubMed**

("Inflammatory Bowel Diseases"[Mesh] OR "inflammatory bowel disease"[tiab] OR "IBD"[tiab] OR "ulcerative colitis"[Mesh] OR "ulcerative colitis"[tiab] OR "Crohn Disease"[Mesh] OR "Crohn disease"[tiab] OR "Crohn's disease"[tiab]) AND("Hydroxymethylglutaryl-CoA Reductase Inhibitors"[Mesh] OR "statin"[tiab] OR "statins"[tiab] OR "3-hydroxy-3-methyl-glutarylCoA reductase inhibitor"[tiab] OR "CS-514"[tiab] OR "simvastatin"[tiab] OR "atorvastatin"[tiab] OR "fluvastatin"[tiab] OR "lovastatin"[tiab] OR "rosuvastatin"[tiab] OR "pravastatin"[tiab] OR "pitavastatin"[tiab]) AND ("Colorectal Neoplasms"[Mesh] OR "colorectal"[tiab] OR "colorectum"[tiab] OR "colon"[tiab] OR "rectal"[tiab] OR "rectum"[tiab]) AND ("neoplasms"[tiab] OR "carcinoma"[tiab] OR "cancer"[tiab] OR "tumor"[tiab] OR "malignancy"[tiab] OR "adenoma"[tiab])

**Embase**

('inflammatory bowel disease'/exp OR 'inflammatory bowel disease':ti,ab OR ibd:ti,ab OR 'ulcerative colitis'/exp OR 'ulcerative colitis':ti,ab OR 'crohn disease'/exp OR 'crohn disease':ti,ab OR "crohn's disease":ti,ab) AND ('hydroxymethylglutaryl coenzyme a reductase inhibitor'/exp OR statin:ti,ab OR statins:ti,ab OR '3-hydroxy-3-methyl-glutarylcoA reductase inhibitor':ti,ab OR cs-514:ti,ab OR simvastatin:ti,ab OR atorvastatin:ti,ab OR fluvastatin:ti,ab OR lovastatin:ti,ab OR rosuvastatin:ti,ab OR pravastatin:ti,ab OR pitavastatin:ti,ab) AND ('colorectal cancer'/exp OR colorectal:ti,ab OR colorectum:ti,ab OR colon:ti,ab OR rectal:ti,ab OR rectum:ti,ab) AND (neoplasm:ti,ab OR neoplasms:ti,ab OR carcinoma:ti,ab OR cancer:ti,ab OR tumor:ti,ab OR malignancy:ti,ab OR adenoma:ti,ab)

**Web of Science**

TS=("inflammatory bowel disease" OR "IBD" OR "ulcerative colitis" OR "Crohn disease" OR "Crohn's disease") AND TS=("statin" OR "statins" OR "3-hydroxy-3-methyl-glutarylCoA reductase inhibitor" OR "CS-514" OR "simvastatin" OR "atorvastatin" OR "fluvastatin" OR "lovastatin" OR "rosuvastatin" OR "pravastatin" OR "pitavastatin") AND TS=("colorectal" OR "colorectum" OR "colon" OR "rectal" OR "rectum") AND TS=("neoplasms" OR "carcinoma" OR "cancer" OR "tumor" OR "malignancy" OR "adenoma")
